# Investigation of Partial Discharge Transformation Characteristics in Polyimide (PI) Insulations under High-Frequency Electric Stress

**DOI:** 10.3390/polym16172450

**Published:** 2024-08-29

**Authors:** Bilal Iqbal Ayubi, Li Zhang, Shengrui Zhou, Yiwei Wang, Liang Zou

**Affiliations:** School of Electrical Engineering, Shandong University, Jinan 250100, China; d2019065@mail.sdu.edu.cn (B.I.A.); 202114618@mail.sdu.edu.cn (S.Z.); 202214650@mail.sdu.edu.cn (Y.W.); zouliang@sdu.edu.cn (L.Z.)

**Keywords:** partial discharge (PD), polyimide (PI), high-frequency insulation, high-frequency power transformers (HFPTs), plasma model

## Abstract

This research delves into the primary issue of polyimide (PI) insulation failures in high-frequency power transformers (HFPTs) by scrutinizing partial discharge development under high-frequency electrical stress. This study employs an experimental approach coupled with a plasma simulation model for a ball–sphere electrode structure. The simulation model integrates the particle transport equation, Poisson equation, and complex chemical reactions to ascertain microscopic parameters, including plasma distribution, electric field, electron density, electron temperature, surface, and space charge distribution. The effect of the voltage polarity and electrical energy on the PD process is also discussed. The contact point plays a pivotal role in triggering partial discharges and culminating in the breakdown of PI insulation. Asymmetry phenomena were found between positive and negative half-cycles by analyzing the PD data stage by stage. A significant number of PDs increased at every stage and the PD amplitude was higher during the negative cycle at the initial stage, but in later stages, the PD amplitude was found to be higher in the positive half-cycle, and scanning electron microscopy (SEM) revealed that the maximum damage occurred near the contact point junction. The simulation results show that the plasma initially accumulates the electron density near the contact point junction. Under the action of the electric field, plasma starts traveling at the PI surface outward from the contact point. Before the PD activity, all parameters have higher values in the plasma head. The microscopic parameters reveal maximum values near the contact point junction, during PD activities where significant damage takes place. These parameter distributions exhibit a decreasing trend over time as when the PD activity ends. The model’s predictions are consistent with the experimental data. The paper lays the foundation for future research in polymer insulation design under high-frequency electrical stress.

## 1. Introduction

The power systems currently under development exhibit novel characteristics, such as the extensive utilization of renewable power sources, integration of large-scale AC-DC grids, and the widespread implementation of power electronic devices in power systems. A power transmission and transformation electrical machine known as a high-frequency power transformer (HFPT), also named a solid-state transformer (SST), has emerged into a power system. HFPTs offer adjustable voltage and current on both sides, controllable power factors, and improved power quality, making them highly applicable in various fields. SST technology finds wide usage in renewable energy systems and distributed energy sources, enhancing the overall efficiency of the power system [1,2].

The insulation system of HFPTs is exposed to high voltage, high frequency, and high temperature, which can cause early insulation failures and aging [3]. Partial discharge is believed to be the primary cause of these insulation failures, gradually degrading the insulation system and ultimately leading to insulation breakdown [4]. Polyimide (PI) is extensively utilized in the inter-turn and ground insulation of HFPTs due to its outstanding dielectric properties, high temperature, and radiation resistance [5,6].

Previous research has explored the phenomena of partial discharge (PD) and aging of insulation materials, highlighting the impact of different factors on polyimide aging and PD mechanisms [7]. the partial discharge (PD) characteristics and failure mechanisms of multilayer polyimide (PI) insulation in solid-state transformers (SST) under high-frequency (HF) electrical-thermal coupling stress. The results reveal that frequencies above 10 kHz produce a distinctive rabbit-ear-like discharge pattern, and higher temperatures reduce PD inception voltage while increasing PD magnitude, accelerating insulation degradation. The research outlines a three-stage failure process of the PI system and proposes a PD development model, offering insights for understanding PD mechanisms in SSTs under HF stresses [8]. The rise time of repetitive square wave voltage significantly influences partial discharge (PD) characteristics, with longer rise times leading to lower magnitude PD pulses and shorter rise times enhancing high-frequency energy components [9]. The study examines under repetitive impulsive voltage waveforms, finding that RPDIV decreases with increasing frequency, then stabilizes, and decreases linearly with pressure [10].

A study analyzed the PD resistance of polymers in power equipment by examining their PD characteristics [11]. Decreasing the pressure and increasing the temperature can significantly influence the insulation damage and PD phenomena [12]. Experimental investigations of the PD characteristics under high-rise rate nanosecond pulse voltage have been conducted, analyzing the effect of nanosecond pulse rise rates on PD characteristics [13].

A study shows PD behavior under different voltage frequencies to understand premature insulation failures and found a correlation between the insulation life and PD [14]. The impact of space charge deposited by PWM pulses on PD behavior is significant at high frequencies [15]. An FEA model was utilized to compute the electric field distribution, which revealed that PD events have a longer duration and a higher true charge magnitude at cruise altitude compared to ground level, but a little delay in PD formation under low-pressure conditions [16].

**Figure 1:**
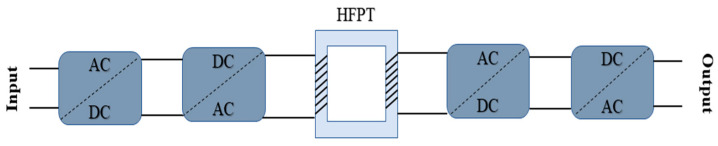
Three-stage SST topologies clearly show the HFPT.

To investigate insulation failure between windings in PETs, a model of twisted pair wires was employed to analyze PD behavior over 50 Hz to 20 kHz. The findings suggest that at high frequencies, the initial discharge voltage and discharge repetition increase while the amplitude of PD reduces [17]. A study explored the mechanisms underlying the changes in PD time lag, magnitude, endurance lifetime, and eroded area by the discharge with pressure through PD and lifetime endurance tests conducted under repetitive pulse voltage and controlled pressure [18]. As the frequency of the applied voltage increases, the amplitude of the electron temperature exhibits an upward trend. Conversely, the surface charge density gradually decreases with the rising frequency. These two factors combine to make partial discharge most severe when the frequency of the applied voltage reaches 15 kHz [19]. The life estimation and degradation of polymeric dielectrics due to partial discharges (PDs) in spherical cavities were investigated using a simulation-based approach. The findings suggest that the degradation rate increases linearly with both the voltage magnitude and frequency, providing valuable insights for prognosis tools in electrical insulation systems [20]. A clear representation of SST topology is shown in Figure 1.

The literature review reveals an ongoing debate on insulation partial discharge phenomena, degradation, and breakdown mechanisms. While a significant portion of the literature is experimental-based, which is not enough to reveal the complete breakdown phenomena, it falls short in elucidating insulation failure mechanisms due to the lack of identification of microscopic parameters such as plasma distribution, electric field distribution, electron density distribution, electron temperature, surface, and space charge distribution. This paper proposes a numerical simulation model based on the finite element method coupled with experimental work to gain a deeper understanding of partial discharge phenomena and breakdown mechanisms of polyimide insulation under high-frequency sinusoidal voltage. A ball–plate electrode structure was employed for both the simulation and experimental work. By analyzing PD mechanisms at high-frequency voltages and utilizing a numerical simulation method, this study provides insights into optimizing polymer insulation used at high frequencies.

## 2. Materials and Methods

### 2.1. Experimental Arrangements

**Figure 2:**
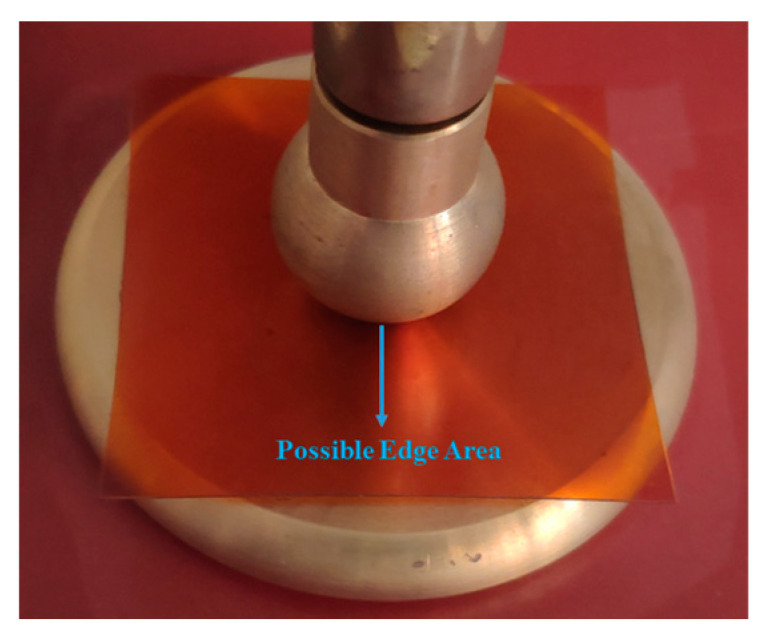
Electrode configuration for PD test.

**Figure 3:**
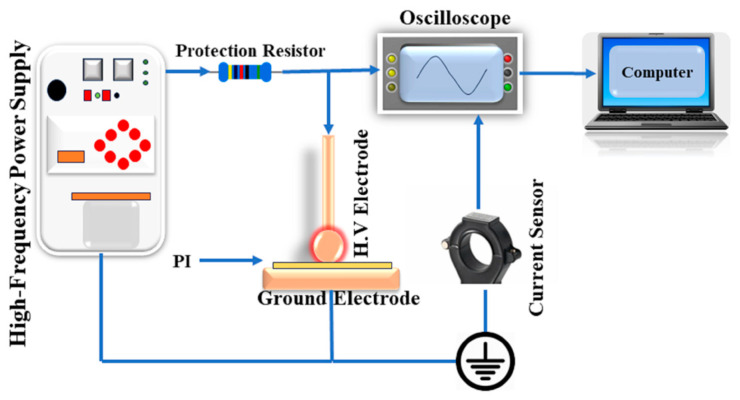
Schematic diagram of partial discharge experimental setup.

In this study, a 75 μm thick polyimide (PI) film with an area of 5 cm × 5 cm was used as the test material. The electrode configuration is presented in Figure 2. The partial discharge test setup was assembled based on the schematic diagram in Figure 3. The ball–plate electrode configuration replicates the non-uniform electric field experienced by the PI in HFPTs. The dimensions of the electrodes align with the international standard IEC 60243-1:2013 “Insulating Materials” [21]. The ball electrode has a diameter of 20 mm, the plate electrode has a thickness of 10 mm, and the plate has a straight diameter of 75 mm.

A sinusoidal voltage with a peak-to-peak value of 0–2 kV and a frequency of 30 kHz was employed. To capture partial discharge signals due to their wide-band and low-amplitude characteristics, the ETS-93686 pulse current sensor was employed. Simultaneously, a Tektronix MDO3024 four-channel oscilloscope, Shanghai, China was utilized to acquire high-frequency sinusoidal and partial discharge signals. Real-time data were transferred to a computer for storage using the USB serial bus and LabVIEW’s data acquisition storage system.

### 2.2. Plasma Simulation Model

Advancements in the simulation of discharges have the critical importance of comprehending the governing equations, chemical reactions, and interactions with dielectric boundaries [22]. To model the partial discharge process, this paper employed a plasma interface that incorporates the migration in the electric field equation, heavy species transport equation, and Poisson equation. An accurate representation of the PD process and an understanding of the solid insulation surface’s influence are both essential aspects of partial discharge modeling. The interactions between particles and the insulation surface lead to secondary electron emission, which must be incorporated into the electron source term using traditional fluid equations. The rate of electron density change is described by the Poisson equation as follows:

(1) $$\frac{\partial }{\partial t}\left(n_{e}\right)+\nabla \cdot \left[-\left(\mu_{e}\cdot E\right)n_{e}-D_{e}\cdot \nabla n_{e}\right]=R_{e}-\left(u\cdot \nabla \right)n_{e}$$

(2) $$\nabla \cdot \left(\varepsilon_{0}\varepsilon_{r}E\right)=\rho_{q}$$

where the electron density *n_e_* is expressed in units of 1/m^3^, while the electron rate expression *R_e_* is measured in 1/(m^3^·s), the mobility of the electron *μ_e_* is measured in m^2^/(V·s), ***E*** is the electric field strength measured in *V/m* and ***E*** = −∇*V* where *V* represents the electric potential. The electron diffusivity ***D****_e_* is measured in m^2^/s, the fluid velocity vector ***u***, *ε*_0_ is the vacuum permittivity, *ε_r_* is the relative permittivity, and *ρ_q_* is the space charge density. However, the local field approximation must be utilized to establish a relationship between the mean electron energy ε and the reduced electric field (*E/N*). The sum of the mass fractions *w* can be calculated using the equation

(3) $$w=1-\sum_{k=1}^{Q}w_{k}$$

where *w_k_* is the mass fraction. If a reacting flow is *k* = 1…, *Q* species. The first species is given by

(4) $$\rho \frac{\partial w}{\partial t}+\rho \left(u\cdot \nabla \right)w_{k}=\nabla \cdot j_{k}+R_{k}$$

where the density of the mixture is denoted by ***ρ***, while ***u*** represents the average mass fluid velocity and *R_k_* is the rate expression for *k*. The diffusive flux vector is ***j****_k_* = *ρw_k_**V**_k_* where ***V**_k_* is the multicomponent diffusion velocity for species *k*. The electrostatic field is computed using the equation

(5) $$-\nabla \cdot \varepsilon_{0}\varepsilon_{r}\nabla V=\rho_{v}$$

(6) $$\rho_{v}=q\left[\sum_{k=1}^{N}Z_{k}n_{k}-n_{e}\right]$$

where *ρ_v_* is the space charge density, which is automatically computed based on the plasma chemistry specified in the model. *Z_k_* is the number of charges carried by the particle *k*, *n_k_* is the density of the particle *k*, *q* is the amount of elementary charge, and *V* is the electric potential. The disappearance of electrons at the wall is attributed to random motions within the mean free path of the wall. This phenomenon is a result of the secondary emission effect. The boundary condition equation for the normal component of the electron flux at the wall is

(7) $$n\cdot \Gamma_{e}=\frac{1-r_{e}}{1+r_{e}}\left[\frac{1}{2}v_{e,th}n_{e}\right]-\left(\sum \gamma_{i}\left(\Gamma_{i}\cdot n\right)+\Gamma_{t}\cdot n\right)$$

where *r_e_* is the reflection coefficient, *ν_e,th_* is the thermal velocity, *γ_i_* is the secondary emission coefficient, *Γ_i_* is the ion flux of species at the wall, *Γ*_t_ is the thermal emission flux, and *n* is the outward normal. Because of surface reactions, ions are lost to the wall in the heavy species

(8) $$r=k^{f}\prod_{k=1}^{Q}c_{k}^{v}$$

(9) kf=γf1−γf2∏σkvk(Γtot)m148RTπMk

The notation *k^f^* is used to represent a heavy particle type. *Γ_tot_* represents the total surface concentration, while *γ_f_* denotes the forward sticking coefficient. *R* represents the gas constant, *T* represents the gas temperature, *σ_k_* represents the potential length, and *v_k_* represents the stoichiometric matrix. The speed of thermal motion of substance *k* is denoted by *√8RT/πM_k_*, where *k* is the Boltzmann constant, *T* is the absolute temperature, and *M* is the particle mass. The surface charge accumulation node tracks how electric charges build up on the surface of a PI due to incoming ions and electrons. This is especially important when the material borders both a plasma model and a charge conservation feature. Without this node, charged particles would escape the simulation and lead to inaccurate results. Mathematically, the following boundary condition is implemented:

(10) $$-n\cdot \left(D_{1}{-D}_{2}\right)=\rho_{s}$$

where *ρ_s_* is the solution of the following distributed ODE on the boundary:

(11) $$\frac{\partial \rho_{s}}{\partial t}=n\cdot J_{i}+n\cdot J_{e}$$

The electric displacement vectors on both sides of the medium insulating surface are ***D***_1_ and ***D***_2_, respectively, and *ρ_s_* is the surface charge density, where ***n****·**J***_i_ is the normal component of the total ion current density on the wall and ***n****·**J***_e_ is the normal component of the total electron current density on the wall. In addition, a periodic boundary condition on the surface charge density is imposed in the extra dimension.

### 2.3. Plasma Chemical Reactions

To accurately model the discharge process, the chemical reactions considered in this research [23,24,25,26] are presented in Table 1, which includes 20 sets of reaction equations and 6 types of particles. Fluid models require the input of transport coefficients and rate coefficients dependent on the electron energy distribution function (EEDF) for accurate discharge modeling. These coefficients are typically derived by solving the electron Boltzmann equation (BE) using collision cross-section data. In this study, the widely applicable BE solver was employed [27]. The model automatically calculates the space charge density based on the type of plasma chemistry.

A comprehensive set of reactions, including ionization, electron attachment, and other chemical reactions, were incorporated into the model. The model adopted the ionization mechanism for electron impact reactions, leading to the formation of secondary electrons. The threshold energy, also known as the energy loss Δε, was incorporated into the model. This study employed the local field approximation, assuming that the transport and source coefficients were accurately parameterized using the reduced electric field (*E/N*). The simulation uses a finite element discretization with linear shape functions to capture PD activities in actual conditions, where the negative values were not considered.

The relative permittivity of polyimide material is *ε_r_* = 3.0. The pressure is set at 1.013 × 10^5^ Pa, and the initial temperature is 25 °C. The initial electron density is 10^6^ m^−3^ and the ion densities are 10^10^ m^−3^.

### 2.4. Mesh for Model Solution

**Figure 4:**
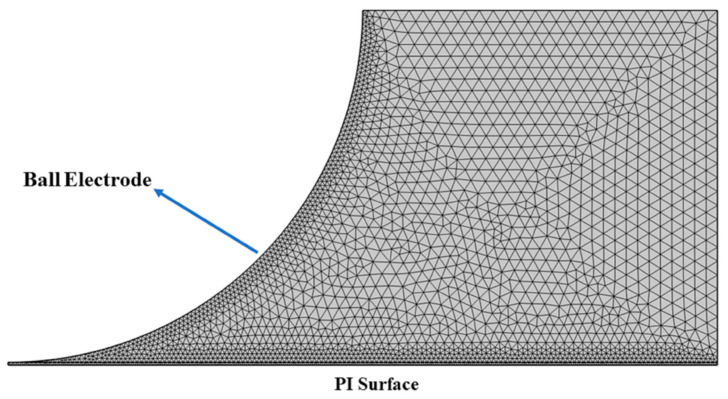
Grid distribution mesh for model.

The effectiveness of plasma simulation in COMSOL is contingent upon several factors, including the size and geometry of the plasma source, the plasma parameters, and the simulation requirements. However, the grid distribution during sinusoidal voltage is dictated by the underlying plasma physics and the phenomena that need to be resolved. The grid distribution employed in this paper is depicted in Figure 4. We simulated the specified affected area, and the grid should be significantly finer near the ball electrode and PI surface, where the plasma density is high and the electric field is stronger. This refinement is essential for capturing the sheath region accurately, a thin layer of plasma adjacent to the ball electrode where the electric field is more intense and the plasma properties can deviate considerably from the bulk. Away from the ball electrode, where the electric field weakens, the grid can be coarser. This paper solved the number of degrees of freedom (grid points) as 28,271.

## 3. Results and Discussion

### 3.1. Partial Discharge Evolution Characteristics Analysis

In this study, a partial discharge (PD) is initiated between the ball electrode and the PI insulation surface. PDs cause localized damage to the insulation, which can accumulate over time and lead to more extensive deterioration. However, understanding PD behavior is complex due to several challenges. These include variations in the height of the air gap between the electrode and insulation, as well as the irregular geometry created by the contact point between them.

**Figure 5:**
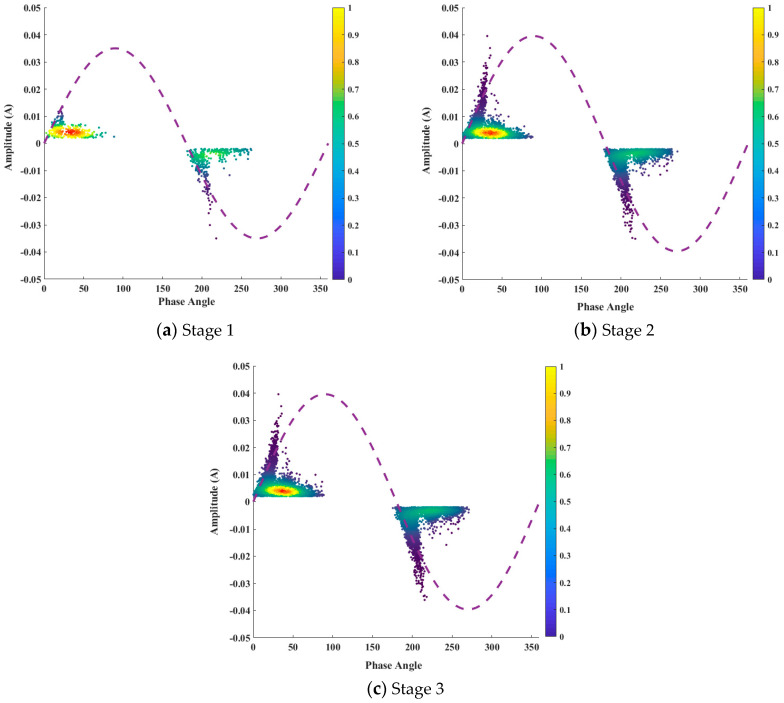
PD characteristics during three stages.

The edge of a metal conductor intensifies the electric field on the surface of PI, increasing the PD formation and insulation breakdown. The PD region is created by applying a voltage between the electrode, air, and polyimide (PI). The higher permittivity of polyimide compared to air caused an increased electric field within the air and insulation surface. This paper divided the PD evolution characteristics into three stages, as shown in Figure 5. The data points in these graphs are color-coded based on the density of PD events. Points with lower PD activity are colored blue, while points with higher activity are colored yellow to red. This color gradient effectively visualizes the variation in the PD density across the data set.

To obtain statistically significant PD features, data from all voltage cycles, including those leading to breakdown, were collected and combined to represent a single cycle. These statistically derived PD data are presented in Table 2.

Stage 1. Partial Discharge Initiation

Figure 5a shows the initial stage of partial discharge (PD) activity, which was observed after an inception voltage of almost 1.3 kV. These voltages represent the minimum threshold required for the initiation of detectable PD events within the polyimide sample under the ball–plate electrode configuration. The electric field within the PI becomes stronger gradually, leading to a higher probability of localized ionization and micro-discharges. The number of recorded PD events, with a total of 706 observed, indicates the formation of discharge channels and propagation of existing ones.

While there are maximum PD amplitude values of 0.013 A and 0.034 A for the positive and negative half-cycles, respectively, an increase in the PDs is observed in the negative half-cycle. This stage exhibits an asymmetry in the discharge intensity with a higher amplitude and the number of PDs during the negative half-cycle. The asymmetry in the discharge intensity and number of discharges is related to the inherent properties of the polyimide and the ball–plate electrode configuration, which could influence the distribution of the electric field during the positive and negative cycles.

Stage 2. PD Propagation

Figure 5b shows a substantial rise in the number of recorded PD events, with 4836 detected in this stage. This represents a significant increase compared to the previous stage, indicating a continuous intensification of the discharge process. The maximum PD amplitude in this stage was measured with values of 0.039 A and 0.034 A for the positive and negative half-cycles, respectively. However, an increase is observed in the amplitude during a positive cycle. An asymmetry in the discharge behavior is observed during both cycles. The number of PD events and amplitude are higher during the positive half-cycle, showing a continued discharge initiation under this polarity. The increase in the number of PD events is attributed to the continued growth and propagation of discharge channels. During this stage, discharges appear like rabbit ears.

Stage 3. Breakdown

Figure 5c shows an exponential rise in the number of recorded PD events, with 8910 detected in this stage. The exponential rise in PD events shows a transition from PD propagation to a breakdown in this stage. This is due to the merging and expansion of existing discharge channels, leading to a more extensive network of localized breakdowns within the PI insulations.

**Figure 6:**
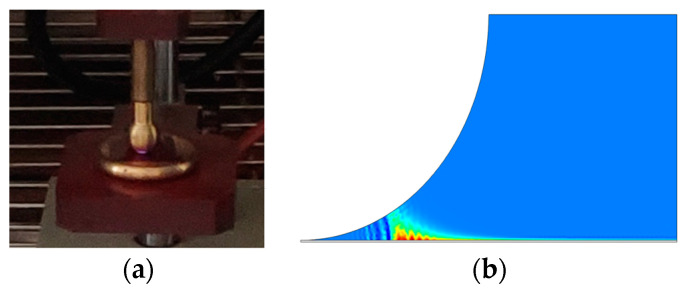
Plasma generation: (**a**) experiment, (**b**) simulation.

The maximum PD amplitude in this stage shows a minimal increase, with values of 0.039 A and 0.036 A for the positive and negative half-cycles, respectively. The minimal increase in the amplitude despite the significant rise in PD events might imply that the individual discharge events are not necessarily becoming more intense, but rather more frequent and widespread throughout the material. An asymmetry in the discharge behavior is still observed with the number of PD events and amplitude remaining higher during the positive half-cycle. During this stage, discharges still appear like rabbit ears. Importantly, this stage is characterized by insulation breakdown within the PI sample. This shows a critical failure point where the insulating properties of the material can no longer withstand the ongoing discharge activity. A plasma spark generation during the experiment and simulation is shown in Figure 6. In Figure 6a, plasma formation is clearly visible below the high-voltage electrode. Similarly, in Figure 6b, the plasma beneath the high-voltage electrode displays a color gradient ranging from red to sky blue

### 3.2. PD Damage Analysis

The contact point where the metal, solid insulator, and gas interface are present often acts as the initial site for discharge initiation [28]. As the discharge progresses, energy release and damage tend to accumulate in this region. As energy is released during the PD event, leading to more pronounced physical and chemical changes in the material, the edge due to the contact point junction leads to higher local electric field strengths, which results in increased electrical stress on the insulation material, making it more susceptible to damage and increasing the discharge activity. So, the insulation weakens at the contact point due to electrical stress, which makes it more damaged near the edge. As the distance from the contact point increases, the length of the air duct also increases. Consequently, for the same applied voltage magnitude, the electric field intensity decreases. Therefore, a higher applied voltage is required to induce breakdown in longer air ducts.

**Figure 7:**
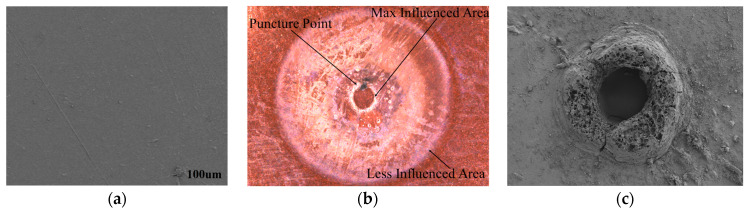
Surface morphologies of PI and high-frequency breakdown of PI: (**a**) SEM of PI, (**b**) complete breakdown affected area, (**c**) SEM of puncture point.

Figure 7 illustrates the state SEM (scanning electron microscopy) of the PI insulation both before the experiment and after the breakdown. In Figure 7a, the surface of the PI is shown to be clean and smooth before the experiment. Figure 7b reveals the complete breakdown, highlighting the most severe damage and the puncture point that occurs near the contact point. The electric field effect diminishes as it moves outward from the contact point, resulting in less damage in these regions. At high frequencies, as depicted in Figure 7c, the film undergoes breakdown, forming a melting region on its surface. The edges of the breakdown area are distinct and smooth, with melting points occurring around this region due to partial discharge erosion. The erosion of the polyimide (PI) films is more pronounced, leading to the formation of a round-shaped melting region.

### 3.3. Evolution of Microscopic Parameters Distribution during Partial Discharge

**Figure 8:**
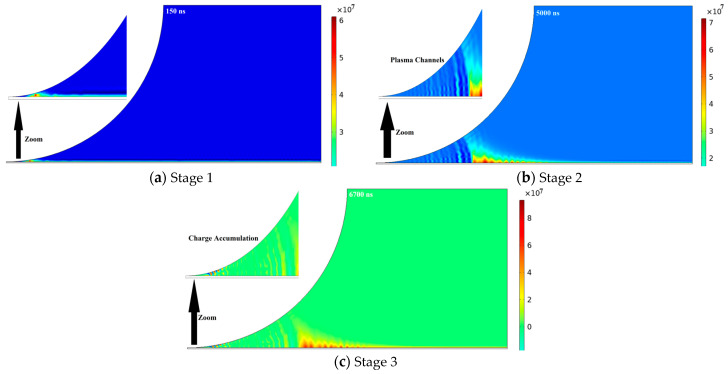
Plasma propagation during three stages.

The contact point junction creates an edge on the surface of the polyimide (PI), which takes part in generating plasma under a high electric field strength. This leads to a complex distribution of plasma (electrons and ions) on the insulation surface. When plasma comes into contact with the surface, it can cause localized discharges. The distribution of plasma has a significant impact on the surface properties of the insulation, altering its chemistry and leading to changes in adhesion. Several factors, such as the gas composition, pressure, surface morphology, and temperature, can influence plasma distribution and the characteristics of plasma discharge [29]. The applied field strength affects the motion of charged particles, resulting in random movements with a peak near the contact point junction. The strong electric field near the electrode ionizes several electrons in the surrounding area, causing the plasma to move away from the electrode due to electric field forces. For analysis of the plasma evolution under sinusoidal voltages on PI, we divided plasma propagation phenomena into three stages, as shown in Figure 8.

Stage 1. Plasma Initiation

During this stage, the electric field of 2.2 × 10^6^ (V/m) starts to ionize surrounding gas molecules in the air and the polyimide (PI) surface near the contact point, as shown in Figure 8a. This means electrons are stripped away from the gas molecules, creating positively charged ions and free electrons. These electrons have an impact on the PI surface, generating more electrons through a secondary electron emission process, which further contributes to the growth of the free electron population. As the ionization and secondary electron emission processes continue, the number of free electrons in the localized region rapidly increases. The electron density reaches 6.1 × 10^7^ (1/m^3^), which indicates the concentration of free electrons at this stage.

Stage 2. Plasma Propagation

The initial plasma formed near the electrode acts as a source of charged particles, which is primarily responsible for the outward expansion of the plasma region. The strong electric field of 6.34 × 10^7^ (V/m) exerts a force on the electrons, further propelling them away from the electrode. As the plasma expands, it creates a partially conductive path between the electrode and the PI surface. This can be visualized as a “plasma channel” where the electric field can now act directly on the plasma, influencing its movement and further ionization processes, as shown in Figure 8b. At this stage, plasma appeared in the shape of a right triangle. Collisions between plasma particles (electrons and ions) within the expanding region can lead to further ionization of neutral gas molecules. This sustains the growth of the plasma and contributes to the observed increase in the electron density 7.15 × 10^7^ (1/m^3^), indicating an increase in the number of free electrons within the expanding plasma compared to the previous stage.

During this stage, charged particles start accumulating in the plasma, which deteriorates the PI surface and causes the PD activity to start. The accumulation of electron density is governed by the properties of the insulating material and the strength of the applied electric field. The electric field induces polarization within the insulating material, causing electrons to be attracted or repelled from the electrode and PI surface, leading to alterations in the local electron density. The accumulation of the electron density near the contact point junction has significant consequences, including the formation of partial discharges and the breakdown of the insulating material.

Stage 3. Partial Discharge activity

A partial discharge event occurs when a localized intense electric field stress forms within the PI material. This event triggers a burst of electrons, causing a surge in the electron density. The high concentration of electrons and strong electric field are likely attributed to activating PD activity on the insulation. The expanding plasma and charge accumulation can lead to a localized region with an extremely strong electric field of 7.47 × 10^7^ (V/m) within the PI material, as shown in Figure 8c. The ionization process leads to a significant surge in the electron density to 9.3 × 10^7^ (1/m^3^), compared to previous stages.

The intense electric field and high electron density cause a localized breakdown of the PI’s insulating properties. This kind of breakdown is “partial” because it does not completely deteriorate the insulating material. However, it can still damage the PI at the breakdown location. During the positive cycle, the PD activity begins at 6700 ns and terminates at 7650 ns. During the negative cycle, a bigger PD activity begins at 22,110 ns and terminates at 22,520 ns.

### 3.4. Electron Density and Electric Field Distribution

**Figure 9:**
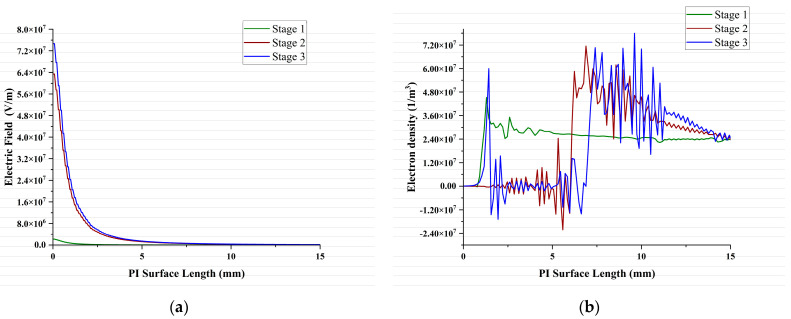
(**a**) Electric field distribution, (**b**) electron density distribution.

The initial high electric field accelerates the free electrons present, causing them to collide with gas molecules and the PI surface. This creates secondary electrons and initiates avalanche ionization, leading to a rapid rise in the electron density. The electric field and electron density stage-by-stage distribution on the PI surface are shown in Figure 9. During stage 1, the electric field remains relatively constant as the initial electron population is low. The electron density is mainly concentrated near the contact point junction. The expanding plasma region and accumulated charges alter the electric field distribution. During stage 2, the electric field becomes more focused near the remaining gap between the plasma and the PI. This stronger electric field, along with secondary processes within the plasma, can further increase the electron density in the plasma head, although the trend shows a continued rise without a distinct peak. The intense electric field within the PI material increases stage by stage and triggers a localized avalanche of ionization events. This rapid increase in free electrons leads to the dramatic surge in the electron density observed. As the distance increased from the ball electrode, the electric field strength had a lowering trend, as shown in Figure 9a. Figure 9b shows that the electron density is at its maximum in the head of the plasma during all stages. Stage 3 also shows the electron density becoming high and accumulating near the contact point junction, which will further be responsible for PD activity.

### 3.5. Surface Charge and Space Charge Distribution

**Figure 10:**
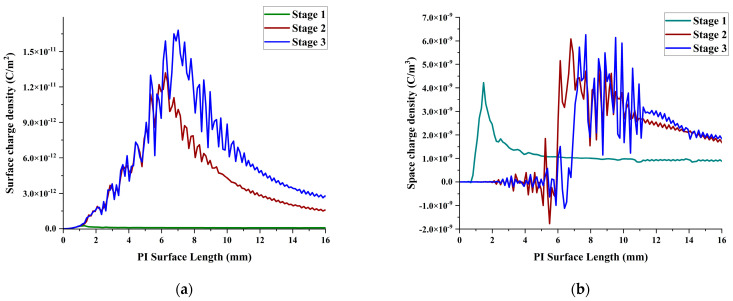
(**a**) Surface charge distribution, (**b**) space charge distribution.

The charge accumulation on the surface of the material changes the distribution of the electric field along the surface and velocity [30], which affects the development of the partial discharge The simulated surface charge density distribution is shown in Figure 10a. During all the stages, most areas of the material surface accumulate positive charge. At the same time, with the development of surface flow, the accumulation of positive surface charge continues to increase, and is at its maximum near the contact point junction. It can be seen that the surface charge accumulation rate increases rapidly during the development stage of PD activity. The surface charge density reaches 16.86 × 10^−12^ (C/m^2^).

The presence of a space charge can influence partial discharge (PD) characteristics. When space charge accumulates near the electrode, it can distort the electric field distribution and increase the local electric field strength [31], leading to an increased PD initiation. Additionally, the accumulated space charge modifies the charge injection at the electrode–insulator interface. Space charge affects the energy distribution of injected electrons and holes, leading to a change in the distribution of PD events. Taking the line parallel to the surface of the insulating material (y = 0.020 mm), the space charge distribution at 0.020 mm above the gas–solid interface can be obtained, see Figure 10b. It can be seen that with the development of along-surface flow, the overall space charge volume is increasing, and it is mainly concentrated in the head of the plasma. Moreover, the positive and negative charge separation phenomenon occurs in the head of the streamer, which is caused by the electric field pulling the positive and negative ions in opposite directions and the different migration rates of ions and electrons. In addition, a negative charge is also found near the electrode, because the ionization near the electrode is intense.

In the early stage of development of the plasma streamer, the electric field strength at the electrode increased with the increase in the applied voltage, and the ionization process intensified, increasing the positive ions. After that, with the continuous development of the plasma channel, the distance between the plasma head and the electrode increases. The contribution of positive charge accumulation near the electrode becomes weaker, and the positive ions generated at the electrode will collide with the electrode quickly under the action of the electric field, disappear, and emit secondary electrons, which further leads to a reduction in the amount of positive charge near the electrode. The space charge at the third stage is 6.2 × 10^−9^ C/m^2^.

### 3.6. Validation of Model

**Figure 11:**
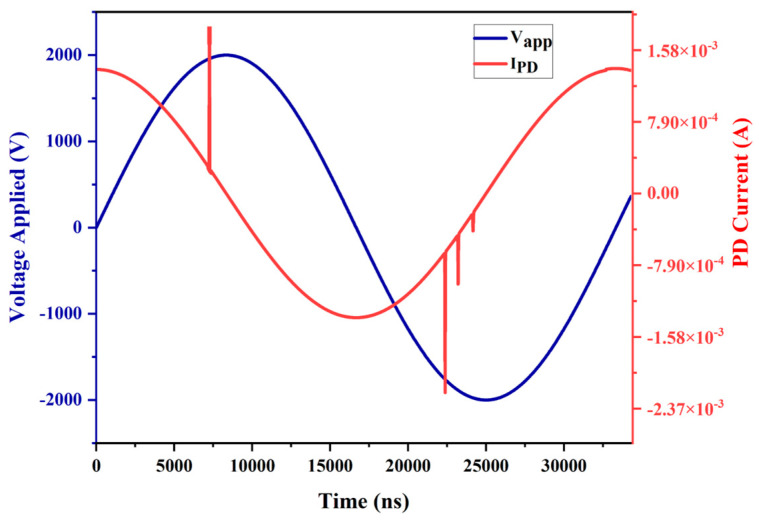
The waveform of simulated PD current vs. input voltage.

To validate the prediction ability of our model, we compared the simulated PD current with the experimental data. The predicted PD current amplitude in our model is in the range of the measured values of the initial stage of the experimental data. This paper considers PD on the positive side during the positive cycle and on the negative side during the negative cycle. Asymmetry phenomena exist both in the simulation results and experimental data, as shown in Figure 11. During the positive half-cycle, the number of PD activities is one, and the overall PD amplitude is 0.002 A. During the negative half-cycle, the number of PD activities is three, and the PD maximum amplitude is 0.0022 A, which also validates our model with the experimental data.

### 3.7. Distribution Parameters during PD Activities

**Figure 12:**
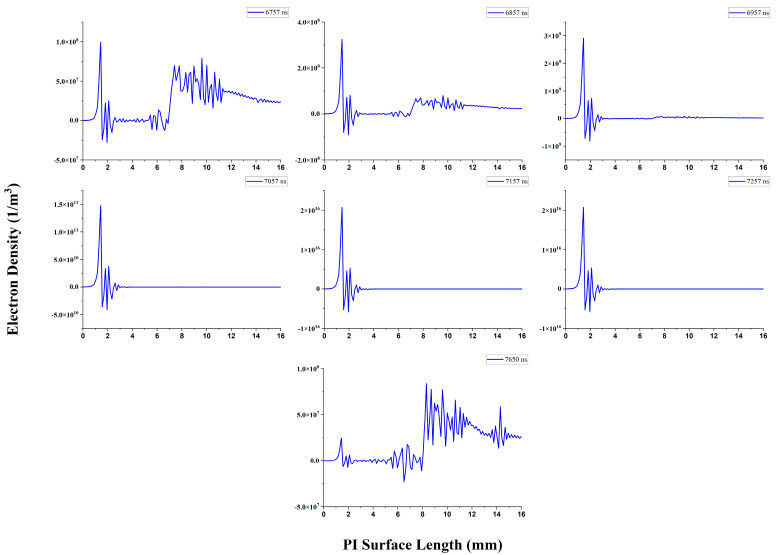
Electron density distribution at different time steps during PD activities.

There are four PD activities during the complete cycle; one PD activity occurs in the positive half-cycle and three PD activities occur in the negative half-cycle. The mechanism behind the PD activity is the same during all PD activities. However, the microscopic parameters and PD amplitude are different. As discussed earlier, the first PD is from 6700 ns to 7650 ns. As shown in Figure 12, it is observed that the electron density starts reducing in the plasma head and increasing near the contact point junction when the PD activity starts. The maximum electron density found at 7257 ns is 3.77 × 10^16^ (1/m^3^); after that, the electron density starts decreasing until the PD activity ends. At 7650 ns, again, the electron density starts increasing in the plasma head and reducing near the contact point junction. For the simplification of the analysis, we explained one PD activity due to the same behavior of PD activities and tabulated all PD activities. It is found that the electron density correlates with the PD amplitude. The simulated PD amplitude is higher at a greater electron density. The electron temperature was maintained at 5.25 V during the PD activity. The PD activity data are shown in Table 3. The surface charge and space charge densities are mixed with positive and negative charges; during the analysis, the adopted charge is near to the contact point junction.

### 3.8. Effect of Voltage Polarity

**Figure 13:**
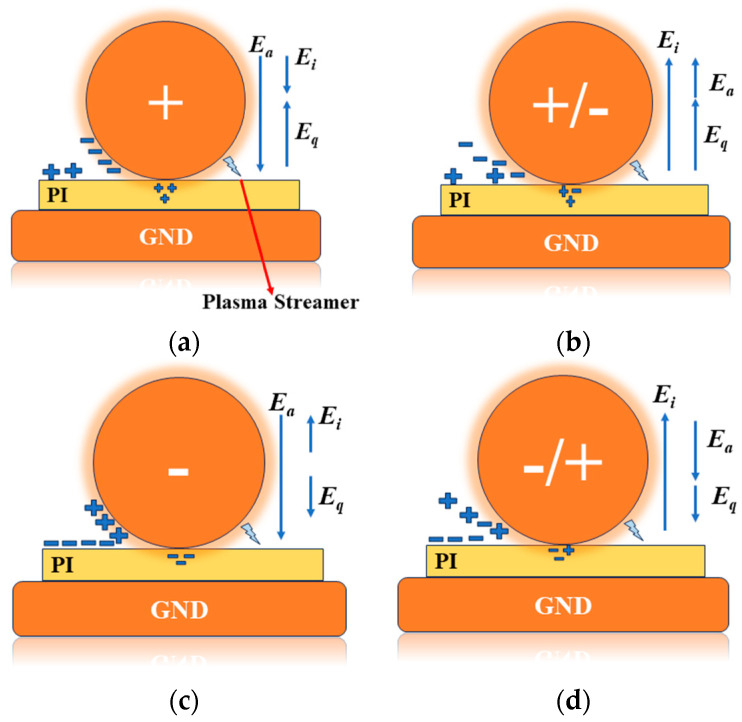
Effect of voltage polarity and charge injection: (**a**) positive cycle, (**b**) negative polarity, (**c**) positive to negative polarity reversal, (**d**) negative to positive polarity reversal.

Partial discharge (PD) in ball–sphere electrode systems primarily occurs in the polyimide film and air gap. During discharge, the ball electrode absorbs charges, which then disperse throughout the space and on the surface. The electric charge distribution and charge injection in Figure 13 depict the effect of the voltage polarity and space charge on the resultant electric field. *E_a_* denotes the applied electric field, *E_q_* denotes the space charge produced by PD, and *E_i_* denotes the resultant electric field produced by *E_a_* and *E_q_* [14]. Charge injection is critical in the behavior of dielectric films, especially when subjected to high electric fields. The process of charge injection involves the transfer of charges (electrons or holes) from metal electrodes into the dielectric material. This process is influenced by several factors, including the strength and polarity of the applied electric field, the properties of the dielectric material, and the nature of the electrode–dielectric interface. Excessive charge injection can lead to localized field enhancement, increased conduction, and eventual breakdown of the dielectric film. Charge injection and subsequent conduction lead to Joule heating, which can further degrade the dielectric material by increasing thermal stress and promoting defect generation [32]. The proposed simulation model focuses on the microscopic parameter’s distribution and partial discharge behavior within polymer insulation materials. While it provides valuable insights into field enhancement and potential degradation paths, the model does not incorporate the effects of Joule heating.

**Figure 14:**
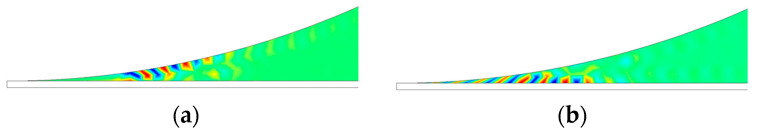
Simulated voltage polarity effect: (**a**) positive half-cycle, (**b**) negative half-cycle.

Under high-frequency electrical stress, PD primarily occurs on the rising edge of the positive and negative half-cycles of the voltage. This is because the electric field between the air gaps rises continuously during the rising phase until it reaches the breakdown field strength, causing the air gap to break down. During PD activity, a large number of charges with the same polarity as the ball electrodes are accumulated on the surface of the insulating medium and space. The simulated effect of polarity on the accumulation of the electron density and surface charge during positive and negative half-cycles is shown in Figure 14, which validates Figure 13a,c. During the positive half-cycle, electron density and charge density are concentrated near the ball electrode, with high electron density indicated in red and low density in blue. In contrast, during the negative half-cycle, the electron density and charge accumulate at the polyimide (PI) surface during the partial discharge (PD) activity.

During the falling phase of the positive and negative half-cycle voltages, the polarity of the charge on the surface of the medium is the same as that in the rising phase, but the weakening effect on the combined field strength is amplified due to the declining normal field strength of the applied electric field. Consequently, the combined field strength between the gaps decreases until it falls below the breakdown field. The voltage polarity reversal effect is shown in Figure 13b,d. Reversing the applied voltage polarity creates a situation where the electric field generated by the accumulated charge on the PI surface opposes the direction of the newly applied field. This opposing field can become the dominant factor during the next discharge event.

### 3.9. Electrical Energy Effect on PD

During partial discharge (PD), localized regions within insulation systems release electrical energy, sometimes manifesting as visible sparks. Understanding the amount of energy discharged is critical for assessing the potential severity and impact on the insulation [33]. Several factors influence this discharge energy: (1) The magnitude of the partial discharge itself plays a direct role. A larger discharge signifies a more significant breakdown in the insulation, leading to a correspondingly greater release of energy. (2) The duration of the partial discharge event also affects the discharge energy. A longer duration allows for a higher cumulative energy release compared to shorter, intermittent discharges. (3) The electric field level at which the partial discharge occurs can influence the discharge energy. Higher electric field strengths tend to result in larger discharge energies due to increased stress on the insulation.

**Figure 15:**
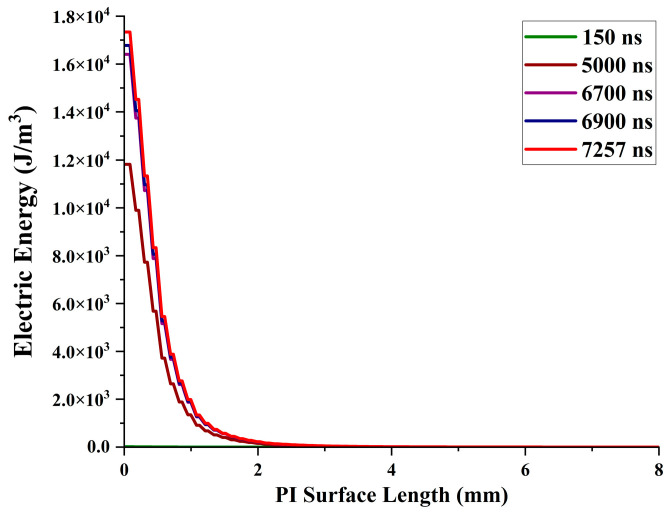
Electrical energy released during PD activity.

Figure 15 represents the stage-by-stage discharge energy and the maximum discharge energy of 17,338 J/m³, which occurs at 7257 ns, representing the peak of the PD activity. This peak suggests a moment of intense stress and potential damage within the insulation. Notably, the discharge energy is highest near the contact point and diminishes as it travels through the material, indicating energy dissipation. Understanding the discharge energy is vital for evaluating the insulation performance and predicting potential failures.

## 4. Conclusions

This research employed a ball–sphere electrode configuration to simulate the partial discharge (PD) process and establish a model for simulating PI insulation surface. The investigation focused on the characteristics of PD transformation under sinusoidal voltage, and microscopic parameters were obtained. The results from the ball–sphere electrode structure revealed several key findings:

The contact point between the electrodes plays a critical role in initiating PD, ultimately leading to the breakdown of the insulating material. PD activity primarily occurs near the rising edges of both positive and negative voltage cycles. This study observed an increasing trend in PD activity throughout the experiment. Interestingly, an asymmetry was identified regarding both the number of PD events and their amplitude. Initially, there were more PDs with a higher amplitude in the negative half-cycle. However, this trend reversed in the later stages, with more frequent and higher-amplitude PDs occurring in the positive half-cycle. The total number of PDs recorded before the breakdown was 8910. The PD amplitude ranged from 0.002 to 0.039 for positive cycles and 0.002 to 0.036 for negative cycles. SEM images revealed significant damage at the contact point and a round melting region on the polyimide film at high frequencies due to partial discharge erosion.

The intense electric field concentrated near the contact point triggers the ionization of electrons in the surrounding area. This creates plasma, which is then pushed away from the electrode due to the repelling forces of the electric field. Interestingly, when PD activity is not yet initiated, the maximum electron density resides within the plasma head, reaching a value of 7.15 × 10^7^ (1/m^3^). However, when starting the PD activity, the electron density spikes near the contact point. The maximum electron density observed during the first PD event is 3.77 × 10^16^ (1/m^3^). This highlights a significant increase in the electron concentration compared to the non-active PD state.

Furthermore, the simulation revealed that microscopic parameters reach their peak values near the contact point junction during the PD activity, the area where the maximum damage was observed. This concurrence suggests a strong correlation between these parameters and the location of the most intense PD activity.

The analysis also indicates the same distribution trend between the electron density and space charge distribution, both within the plasma head and during the PD activity. Notably, the results demonstrate that the amplitude of PD events directly relates to the electron density. It is worth mentioning that the electron temperature remained constant at 5.25 V throughout all PD activities. Finally, the maximum electrical discharge energy recorded during the first PD activity reached a significant value of 17,338 J/m^3^.

## Figures and Tables

**Table 1:** Set of chemical reactions.

**No.**	**Formula**	**Type**	**Reaction Rate k**	**Energy Loss** Δε
R_1_	N_2_ + e => N_2_^+^ + 2e	Ionization	f (e)	15.6 eV
R_2_	O_2_ + e => O_2_^+^ + 2e	Ionization	f (e)	12.06 eV
R_3_	2O_2_ + e => O_2_ + O_2_^−^	Attachment		2 × 10^−45^ × (300/plas.Te)
R_4_	O_4_^+^ + e => 2O_2_	Attachment		1.42 × 10^−14^ × (300/plas.Te)^−0.5^
R_5_	O_2_^+^ + e => 2O	Attachment		2 × 10^−15^ × (300/plas.Te)
R_6_	N_2_^+^ + N_2_ + e => 2N_2_	Attachment		6.07 × 10^−36^ × (plas.Te^−2.5^)
R_7_	N_2_^+^ + 2e => N_2_ + e	Attachment		5.651 × 10^−48^ × (plas.Te^−0.8^)
R_8_	N_2_^+^ + N_2_ + O_2_ => N_4_^+^ + O_2_	Reaction		5 × 10^−45^
R_9_	N_2_^+^ + N_2_ + N_2_ => N_4_^+^ + N_2_	Reaction		5 × 10^−45^
R_10_	N_4_^+^ + O_2_ => O_2_^+^ + 2N_2_	Reaction		2.5 × 10^−20^
R_11_	N_2_^+^ + O_2_ => O_2_^+^ + N_2_	Reaction		6 × 10^−31^
R_12_	O_2_^+^ + O_2_ + O_2_ => O_4_^+^ + O_2_	Reaction		2.4 × 10^−46^
R_13_	O_2_^+^ + O_2_ + N_2_ => O_4_^+^ + N_2_	Reaction		2.4 × 10^−46^
R_14_	O_4_^+^ + O_2_^−^ => 3O_2_	Reaction		1.0 × 10^−17^
R_15`_	O_4_^+^ + O_2_^-^ + O_2_ => 3O_2_ + O_2_	Reaction		2.0 × 10^−41^
R_16_	O_4_^+^ + O_2_^-^ + N_2_ => 3O_2_ + N_2_	Reaction		2.0 × 10^−41^
R_17_	O_2_^+^ + O_2_^-^+O_2_ => 2O_2_ + O_2_	Reaction		2.0 × 10^−41^
R_18_	O_2_^+^ + O_2_^-^ + N_2_ => 2O_2_ + N_2_	Reaction		2.0 × 10^−41^
R_19_	O + O_2_ + O_2_ => O_3_ + O_2_	Reaction		5.651 × 10^−34^
R_20_	O + O_2_ + N_2_ => O_3_ + N_2_	Reaction		5.651 × 10^−34^

**Table 2:** PD statistical data.

No.	Total No. of PDs	No. of PDs Positive Cycle	No. of PDs Negative Cycle	Positive Cycle PD Amp	Negative Cycle PD Amp
Stage.1	706	349	357	0.002−0.013	0.002−0.034
Stage.2	4836	2514	2322	0.002−0.039	0.002−0.034
Stage.3	8910	4709	4201	0.002−0.039	0.002−0.036

**Table 3:** PD activity data.

No.	Time (ns)	PD Amp (V)	Electric Field Strength (V/m)	Electron Density (1/m^3^)	Electron Temp (V)	Surface Charge (C/m^2^)	Space Charge (C/m^3^)
PD_1_	6700–7650	0.002	7.68 × 10^7^	3.77 × 10^16^	5.25	−3.2 × 10^−5^	−4.1 × 10^−3^
PD_2_	22,110–22,560	0.0022	6.5 × 10^7^	9.14 × 10^16^	5.25	−13.5 × 10^−5^	−1.8 × 10^−4^
PD_3_	22,900–23,450	0.001	7.68 × 10^7^	3.17 × 10^16^	5.25	−2.2 × 10^−4^	−3.6 × 10^−3^
PD_4_	23,700–24,490	0.0005	7.38 × 10^7^	1.52 × 10^16^	5.25	−1.9 × 10^−4^	−1.5 × 10^−3^

## Data Availability

Data are available upon request.
